# Supplementary material for: Tead1a Initiates Transcriptional Priming Through the TEAD1a/YAP‐Notch1‐Spi1/Cebpα Axis to Promote Neutrophil Fate
Source: Adv Sci (Weinh). 2025 Aug 28;12(41):e05441. doi: 10.1002/advs.202505441 (PMC12591127; doi:10.1002/advs.202505441)
Supplement: Supplementary file 4 — Supplementary Dataset [file ADVS-12-e05441-s004.zip › 3-4.zip!/3-4/02-4.cluster/cluster10/Enrich/ego_CC.pdf]

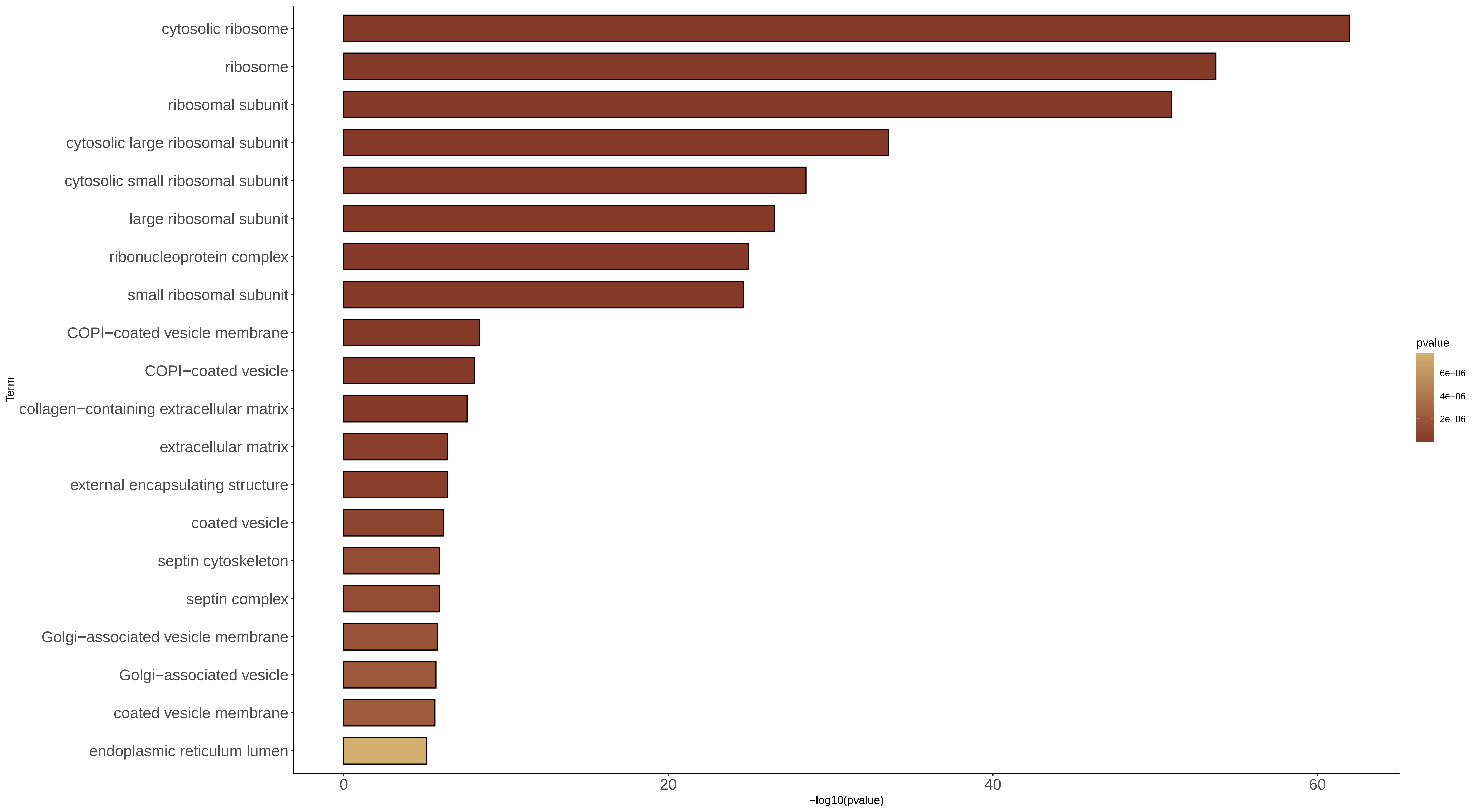

cytosolic ribosome
ribosome
ribosomal subunit
cytosolic large ribosomal subunit
cytosolic small ribosomal subunit
large ribosomal subunit
ribonucleoprotein complex
small ribosomal subunit
COPI−coated vesicle membrane
COPI−coated vesicle
collagen−containing extracellular matrix
extracellular matrix
external encapsulating structure
coated vesicle
septin cytoskeleton
septin complex
Golgi−associated vesicle membrane
Golgi−associated vesicle
coated vesicle membrane
endoplasmic reticulum lumen
Term
0
20
40
60
−log10(pvalue)
pvalue
6e−06
4e−06
2e−06
